# The predictive value of combined assessment of estimated glucose disposal rate and non-high-density lipoprotein cholesterol to high-density lipoprotein cholesterol ratio for cardiovascular disease risk: a nationwide cohort study

**DOI:** 10.3389/fmed.2026.1828999

**Published:** 2026-05-29

**Authors:** Qingfang Meng, Zheng Zhang, Longyan Lv, Xingchen Wang, Lei Wang, Lin Ji, Xia Li, Chong Ma, Mingqing Zhang

**Affiliations:** 1 Department of Neurology, Second Affiliated Hospital of Shandong University of Traditional Chinese Medicine, Jinan, Shandong, China; 2 Department of Emergency, Second Affiliated Hospital of Shandong University of Traditional Chinese Medicine, Jinan, Shandong, China; 3 First Clinical Medical College, Shandong University of Traditional Chinese Medicine, Jinan, Shandong, China

**Keywords:** cardiovascular diseases, cohort study, eGDR, insulin resistance, NHHR, risk stratification

## Abstract

**Background:**

Insulin resistance and dyslipidemia are important risk factors for cardiovascular disease (CVD). The estimated glucose disposal rate (eGDR) and the non-high-density lipoprotein cholesterol to high-density lipoprotein cholesterol ratio (NHHR) are effective indicators for assessing insulin sensitivity and the degree of atherosclerosis, respectively. However, the combined effects of different combinations of these two parameters on CVD risk remain unclear.

**Methods:**

This study included 6,170 participants with no CVD at baseline, utilizing data from the China Health and Retirement Longitudinal Study (CHARLS). After binary classification of eGDR (median = 10.09) and NHHR (median = 2.81), participants were cross-classified into four groups: high eGDR and low NHHR, high eGDR and high NHHR, low eGDR and low NHHR, and low eGDR and high NHHR. Cox proportional hazards models were used to evaluate the relationship between each group and CVD risk, and the interaction between eGDR and NHHR was further analyzed.

**Results:**

During a median follow-up period of 9 years, 1,963 new cardiovascular events in total were recorded. After multivariate adjustment, in contrast to the control group, individuals with low eGDR and high NHHR had the highest CVD risk (HR = 1.78, 95% CI 1.57–2.00), followed by individuals with low eGDR and low NHHR (HR = 1.56, 95% CI 1.36–1.78). In contrast, the combination of high eGDR and high NHHR was not significantly associated with risk (HR = 1.08, 95% CI 0.94–1.25). Cardiovascular disease risk increased significantly with the number of adverse metabolic states (i.e., low eGDR and/or high NHHR). Neither multiplicative nor additive interaction between eGDR and NHHR reached statistical significance.

**Conclusion:**

Insulin resistance is an independent and potent risk factor for CVD, even in the absence of significant atherogenic dyslipidemia. Combined assessment of eGDR and NHHR can effectively identify high-risk individuals with a “dual metabolic burden” and those with isolated insulin resistance who are potentially at high risk, providing a simple and practical tool for improving CVD risk stratification and prevention.

## 1 Introduction

With a steadily rising burden, cardiovascular disease (CVD) is the primary cause of death and disability across the globe (1, 2). So to prevent CVD, it is necessary to find important modifiable risk factors and optimize risk stratification strategies. Insulin resistance plays an important role in the pathophysiology of CVD through metabolic disorders, inflammatory activation, and endothelial dysfunction (3, 4). Several alternative surrogate markers of insulin resistance have been proposed, such as the triglyceride-glucose (TyG) index and its obesity-related derivatives (TyG-BMI, TyG-WC, TyG-WHtR), the triglyceride to high-density lipoprotein cholesterol ratio (TG/HDL-C), and the estimated glucose disposal rate (eGDR). The TyG index has been validated as a simple, cost-effective, and reliable proxy for insulin resistance associated with increased risk of metabolic syndrome, type 2 diabetes, and CVD (5). Similarly, the TG/HDL-C ratio has recently been shown to be independently associated with arterial stiffness in individuals with prediabetes, suggesting its potential as a simple, cost-effective tool for cardiovascular risk assessment (6). The estimated glucose disposal rate (eGDR), which is composed of glycemic control (HbA1c), history of hypertension, and waist circumference, is a simple and reliable clinical alternative indicator for assessing insulin sensitivity (or insulin resistance status) (7). There is substantial evidence linking increased CVD risk to lower eGDR levels (8–11). Notably, a recent large-scale study in the Chinese population compared eight insulin resistance-related biomarkers, showing that eGDR had the best predictive performance for CVD (especially stroke) across all glycemic states; for a one-standard-deviation increase in eGDR, the risks of heart disease and stroke decreased by 21 and 14.2%, respectively (12). Another independent study compared ten insulin resistance indicators, confirming that eGDR and CVAI were superior to all other surrogate indicators. Specifically, a one-standard-deviation increase in eGDR corresponded to a 17.8% decrease in CVD risk (OR = 0.822, 95% CI 0.696–0.969), and individuals in the highest eGDR quartile had a 47.3% lower risk compared with those in the lowest quartile (13). This remarkable predictive ability likely reflects that eGDR integrates multiple metabolic indicators—including waist circumference, hypertension, and glycemic control (HbA1c)—that collectively influence cardiovascular risk, acting through pathways beyond those covered by TyG or TG/HDL-C. Nevertheless, TyG and TG/HDL-C remain important and convenient screening tools, especially in regions with limited resources and difficulty in obtaining comprehensive metabolic data. Therefore, in this study, we chose eGDR as an indicator of insulin sensitivity. Another known cause of CVD is dyslipidemia, particularly increased atherogenic lipoproteins and decreased high-density lipoprotein cholesterol (HDL-C) (14, 15). Non-HDL-C (which comprises all the cholesterol in atherogenic lipoproteins) to HDL-C ratio (NHHR) can more accurately represent lipid homeostasis (between pro- and anti-atherogenic factors) than individual lipid parameters and has been shown to be a potent predictor of CVD (16, 17).

In terms of pathophysiology, dyslipidemia and insulin resistance are closely related. For example, insulin resistance causes a typical atherosclerotic lipid profile by increasing the secretion of very low-density lipoproteins (VLDL) and inhibiting lipoprotein lipase activity, which results in increased triglycerides and decreased HDL-C (18, 19). The effect of their combined patterns on CVD events at the population level has not yet been completely clarified, although both eGDR and NHHR are intrinsically linked and independently associated with CVD risk. Different combinations of lipid profiles and insulin sensitivity are frequently seen in clinical practice: some patients have dyslipidemia but are insulin-sensitive, while others are insulin-resistant but have lipid levels that are comparatively normal.

This study analyzed data from the nationally representative Chinese Health and Retirement Longitudinal Study (CHARLS) cohort to examine the link between different combinations of eGDR and NHHR and the risk of incident CVD. We hypothesized that individuals exhibiting both low eGDR and high NHHR (the “dual metabolic burden”) would possess a higher risk of CVD than those with a single risk factor. Furthermore, we wanted to find out if the combined evaluation could reveal subgroups with varying risk levels that were not visible with just one indicator.

## 2 Methods

### 2.1 Research design and population

**Figure 1:**
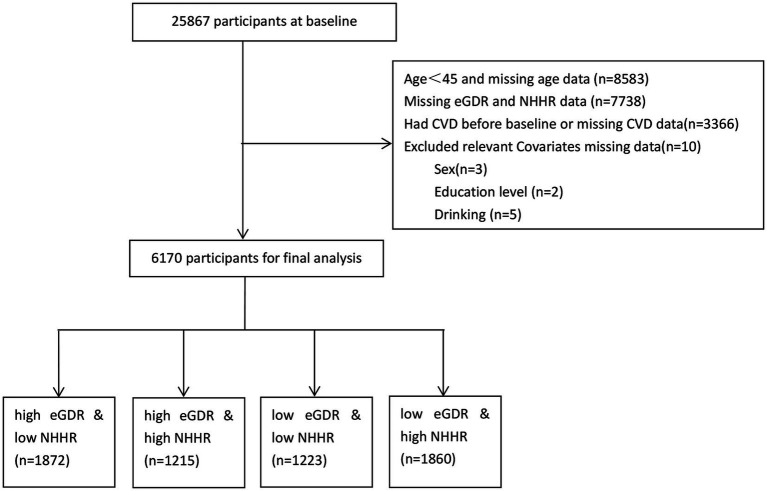
Flowchart of study participant selection. CVD, cardiovascular disease; eGDR, estimated glucose disposal rate; NHHR, non-high-density lipoprotein cholesterol to high-density lipoprotein cholesterol ratio.

The subjects of this study were Chinese community members aged 45 and above from CHARLS, a nationally representative prospective cohort study (20). We used the baseline survey (the first survey in 2011) and follow-up data up to 2020 (the fourth survey). The baseline survey included 25,867 participants. We sequentially excluded individuals who were below the age of 45 or with missing age (*n* = 8,583), those lacking data required for calculating eGDR or NHHR (*n* = 7,738), those with a self-reported baseline history of heart disease or stroke (*n* = 3,366), and those with missing covariate information (gender, education level, alcohol consumption) (*n* = 10). Ultimately, we obtained 6,170 participants (Figure 1). The CHARLS study complied with the ethical principles of the Declaration of Helsinki and received ethical approval from the Ethics Review Committee of Peking University (IRB00001052-11015); in addition, each participant’s voluntary written informed consent was obtained prior to data collection.

### 2.2 Outcome determination and follow-up

The primary outcome was the first occurrence of a CVD event. CVD was determined through self-reported physician diagnosis in a questionnaire: “Has a doctor ever told you that you have been diagnosed with a stroke?” and “Has a doctor ever told you that you have been diagnosed with heart disease, such as heart attack, coronary heart disease, angina, congestive heart failure, or other heart problems?” If any question was answered “yes,” the participant was classified as having experienced a CVD event. Follow-up was initiated at the baseline survey (2011–2012) and continued until the date of the occurrence of a first CVD event, the last interview in the 2020 survey, or the date of loss to follow-up, whichever came first.

### 2.3 Exposure variables: eGDR, NHHR, and their combination

eGDR was calculated using the following validated formula (21, 22): eGDR = 21.158−(0.09 × waist circumference [cm])−(3.407 × hypertension [yes = 1/no = 0])−(0.551 × glycated hemoglobin [%]).

NHHR was calculated using the following formula (23): (total cholesterol−HDL-C)/HDL-C.

Due to the lack of consistent clinical cutoff values for eGDR and NHHR in the Chinese population and the purpose of investigating the relative risks of the combined model, we dichotomized both indicators using the median. This method created balanced combined exposure groups—high eGDR (≥10.09) and low eGDR (<10.09); low NHHR (<2.81) and high NHHR (≥2.81)—which were then cross-classified to create four mutually exclusive exposure groups: the high eGDR and low NHHR group, the high eGDR and high NHHR group, the low eGDR and low NHHR group, and the low eGDR and high NHHR group.

### 2.4 Covariates

Baseline covariates were collected through standardized questionnaires, including (1) demographic characteristics: age, gender, marital status, and educational attainment (illiterate, ≤primary school, ≥secondary school); (2) lifestyle factors: smoking status (never/former/current), drinking status (yes/no); (3) anthropometric and clinical indicators: body mass index (BMI, kg/m^2^), systolic blood pressure (SBP), and diastolic blood pressure (DBP); (4) medical history: defined dyslipidemia (self-reported diagnosis or lipid-lowering drug use), diabetes (diagnosed by the following predefined glycemic criteria: fasting blood glucose equal to or exceeding 7.0 millimoles per liter; a glycated hemoglobin (HbA1c) level of 6.5 percent or higher; self-reported diagnosis; or use of glucose-lowering agents) (24), and hypertension (defined by blood pressure ≥140/90 mmHg, self-reported diagnosis, or antihypertensive drug use) (25, 26); (5) laboratory indicators: total cholesterol (TC), high-density lipoprotein cholesterol (HDL-C), low-density lipoprotein cholesterol (LDL-C), C-reactive protein (CRP), uric acid (UA), and cystatin C (CysC).

### 2.5 Statistical analysis

Continuous variables were expressed as mean ± standard deviation (SD) or median (interquartile range), and categorical variables as frequencies and percentages. Differences between groups were evaluated using the Kruskal-Wallis test or the chi-square test, depending on the type of data. Cumulative CVD incidence was visualized with Kaplan–Meier curves. After adjusting for multiple potential confounding factors, the Cox proportional hazards regression model was used to estimate the hazard ratios (HRs) and 95% confidence intervals (CIs) for the association between combined exposure groups and CVD risk to reveal the risk differences between different exposure combinations. We constructed stepwise adjusted nested models: a baseline model for age and gender (Model 1); a model adding sociodemographic (marital status and education) and lifestyle factors (smoking, and drinking) (Model 2); and a fully adjusted model additionally incorporating BMI, CRP, UA, and CysC (Model 3). The results of Model 3 were reported as the primary finding. Furthermore, the four combined groups were tested for trends as ordinal categorical variables.

To examine the interaction between eGDR and NHHR on CVD risk, we performed two tests: (1) multiplicative interaction: a product interaction term of eGDR and NHHR categorical variables was included in the Cox model (Model 3); (2) additive interaction: the relative excess risk due to interaction (RERI), attributable proportion (AP), synergy index (SI), and their 95% CIs were estimated to assess whether the joint effect of both exposures was greater than the sum of their independent effects.

To test the consistency of the primary association, we repeated the Cox regression analysis (based on Model 3) in different subgroups (including gender, age, marital status, education level, alcohol consumption, smoking, BMI, hypertension, and diabetes status) and calculated the *p*-value for interaction to assess effect modification. To validate the robustness of the results, we performed the following sensitivity analyses: (1) using the complete-case dataset (without multiple imputation); (2) excluding participants who used glucose-lowering, antihypertensive, or lipid-lowering drugs at baseline; and (3) excluding participants with < 2 years of follow-up.

To evaluate the improvement in risk stratification of CVD by the combined model (eGDR+NHHR) compared with single indicators, the Net Reclassification Improvement (NRI) and the Integrated Discrimination Improvement (IDI) were calculated at the 9-year mark (the median follow-up time). The continuous NRI avoids arbitrary risk thresholds. We estimated 95% confidence intervals and *p* values using bootstrap resampling (500 replicates).

All statistical analyses were performed using R software (version 4.2.2). A two-tailed *p*-value of less than 0.05 indicated statistical significance.

## 3 Results

### 3.1 Baseline characteristics

The baseline characteristics of 6,170 participants are shown in Table 1. 45.2% were men, and the average age was 58.5 ± 8.7 years. Clinical indicators, medical history, lifestyle, and demographics all showed significant differences across the four groups (all *p* values < 0.05). From the most favorable metabolic profile (the high eGDR and low NHHR group) to the most unfavorable profile (the low eGDR and high NHHR group), there was a distinct gradient of increasing CVD risk among the four groups. The efficacy of this classification in distinguishing between various metabolic risk levels was validated by the gradual rise in BMI, blood pressure, and CRP levels, as well as by the increasing prevalence of diabetes, dyslipidemia, and hypertension that accompanied this gradient feature.

**Table 1:** Baseline characteristics of participants.

Variables	Total	High eGDR and low NHHR	High eGDR and high NHHR	Low eGDR and low NHHR	Low eGDR and high NHHR	P value
Number of participants	6,170	1872	1,215	1,223	1860	
Age (years)	58.5 ± 8.7	57.2 ± 8.5	57.1 ± 8.2	61.1 ± 9.4	59.0 ± 8.3	< 0.001
Gender, *n* (%)						0.002
Male	2,787 (45.2)	904 (48.3)	564 (46.4)	523 (42.8)	796 (42.8)	
Female	3,383 (54.8)	968 (51.7)	651 (53.6)	700 (57.2)	1,064 (57.2)	
Marital status, *n* (%)						< 0.001
Married	5,570 (90.3)	1716 (91.7)	1,113 (91.6)	1,049 (85.8)	1,692 (91)	
Others	600 (9.7)	156 (8.3)	102 (8.4)	174 (14.2)	168 (9)	
Education level, *n* (%)						< 0.001
Illiteracy	1777 (28.8)	509 (27.2)	322 (26.5)	411 (33.6)	535 (28.8)	
Elementary school or lower	2,548 (41.3)	792 (42.3)	491 (40.4)	511 (41.8)	754 (40.5)	
Middle school and above	1845 (29.9)	571 (30.5)	402 (33.1)	301 (24.6)	571 (30.7)	
Drinking status, *n* (%)						< 0.001
No	3,813 (61.8)	1,104 (59)	818 (67.3)	730 (59.7)	1,161 (62.4)	
Yes	2,357 (38.2)	768 (41)	397 (32.7)	493 (40.3)	699 (37.6)	
Smoking status, *n* (%)						< 0.001
Never smoker	3,841 (62.3)	1,118 (59.7)	743 (61.2)	773 (63.2)	1,207 (64.9)	
Former smoker	476 (7.7)	112 (6)	98 (8.1)	98 (8)	168 (9)	
Current smoker	1853 (30.0)	642 (34.3)	374 (30.8)	352 (28.8)	485 (26.1)	
BMI,kg/m2	23.2 (20.9, 25.8)	21.4 (19.7, 23.3)	22.5 (20.8, 24.3)	23.3 (21.2, 26.1)	25.9 (23.5, 28.1)	< 0.001
SBP, mmHg	130.0 ± 23.7	117.0 ± 12.4	118.5 ± 11.8	143.6 ± 27.2	141.5 ± 24.9	< 0.001
DBP, mmHg	75.5 ± 12.1	69.3 ± 9.1	70.6 ± 8.8	80.9 ± 12.0	81.5 ± 12.2	< 0.001
Dyslipidemia, *n* (%)						< 0.001
No	5,678 (92.0)	1823 (97.4)	1,140 (93.8)	1,123 (91.8)	1,592 (85.6)	
Yes	492 (8.0)	49 (2.6)	75 (6.2)	100 (8.2)	268 (14.4)	
Hypertension, *n* (%)						< 0.001
No	3,828 (62.0)	1856 (99.1)	1,201 (98.8)	239 (19.5)	532 (28.6)	
Yes	2,342 (38.0)	16 (0.9)	14 (1.2)	984 (80.5)	1,328 (71.4)	
Diabetes, *n* (%)						< 0.001
No	5,173 (83.8)	1744 (93.2)	1,065 (87.7)	1,001 (81.8)	1,363 (73.3)	
Yes	997 (16.2)	128 (6.8)	150 (12.3)	222 (18.2)	497 (26.7)	
TC, mg/dL	194.1 ± 38.6	179.5 ± 32.3	205.4 ± 38.3	183.4 ± 32.8	208.3 ± 40.8	< 0.001
HDL-C, mg/dL	51.4 ± 15.2	60.9 ± 13.8	43.2 ± 9.6	60.9 ± 13.8	41.0 ± 9.4	< 0.001
LDL-C, mg/dL	116.5 ± 34.9	104.3 ± 26.2	129.9 ± 36.6	106.1 ± 26.9	126.8 ± 39.5	< 0.001
CRP, mg/L	1.0 (0.5, 2.0)	0.7 (0.4, 1.5)	0.9 (0.5, 1.7)	0.9 (0.5, 2.0)	1.4 (0.8, 2.7)	< 0.001
UA, mg/dL	4.4 ± 1.2	4.1 ± 1.1	4.4 ± 1.2	4.4 ± 1.2	4.7 ± 1.3	< 0.001
CysC, mg/L	1.0 ± 0.2	1.0 ± 0.2	1.0 ± 0.2	1.0 ± 0.2	1.0 ± 0.2	< 0.001
eGDR	9.4 ± 2.3	11.4 ± 0.9	11.2 ± 1.0	7.7 ± 1.3	7.3 ± 1.6	< 0.001
NHHR	3.1 ± 1.7	2.0 ± 0.5	3.9 ± 1.3	2.1 ± 0.5	4.3 ± 2.1	< 0.001
2020 Heart disease, *n* (%)						< 0.001
No	4,775 (93.8)	1,561 (95.2)	988 (94.8)	885 (91.8)	1,341 (92.9)	
Yes	314 (6.2)	78 (4.8)	54 (5.2)	79 (8.2)	103 (7.1)	
2020 Stroke, *n* (%)						< 0.001
No	5,450 (97.3)	1731 (97.9)	1,117 (97.9)	1,043 (97.8)	1,559 (95.9)	
Yes	153 (2.7)	38 (2.1)	24 (2.1)	24 (2.2)	67 (4.1)	
2020 CVD, *n* (%)						< 0.001
No	4,207 (68.2)	1,425 (76.1)	906 (74.6)	757 (61.9)	1,119 (60.2)	
Yes	1963 (31.8)	447 (23.9)	309 (25.4)	466 (38.1)	741 (39.8)	

BMI, body mass index; CRP, C-reactive protein; CVD, cardiovascular disease; CysC, cystatin C; DBP, diastolic blood pressure; eGDR, estimated glucose disposal rate; HDL-C, High-density lipoprotein cholesterol; LDL-C, low-density lipoprotein cholesterol; NHHR, non-high-density lipoprotein cholesterol to high-density lipoprotein cholesterol ratio; SBP, systolic blood pressure; TC, total cholesterol; UA, uric acid.

### 3.2 Association of eGDR and NHHR with CVD risk: individual and joint effects

**Figure 2:**
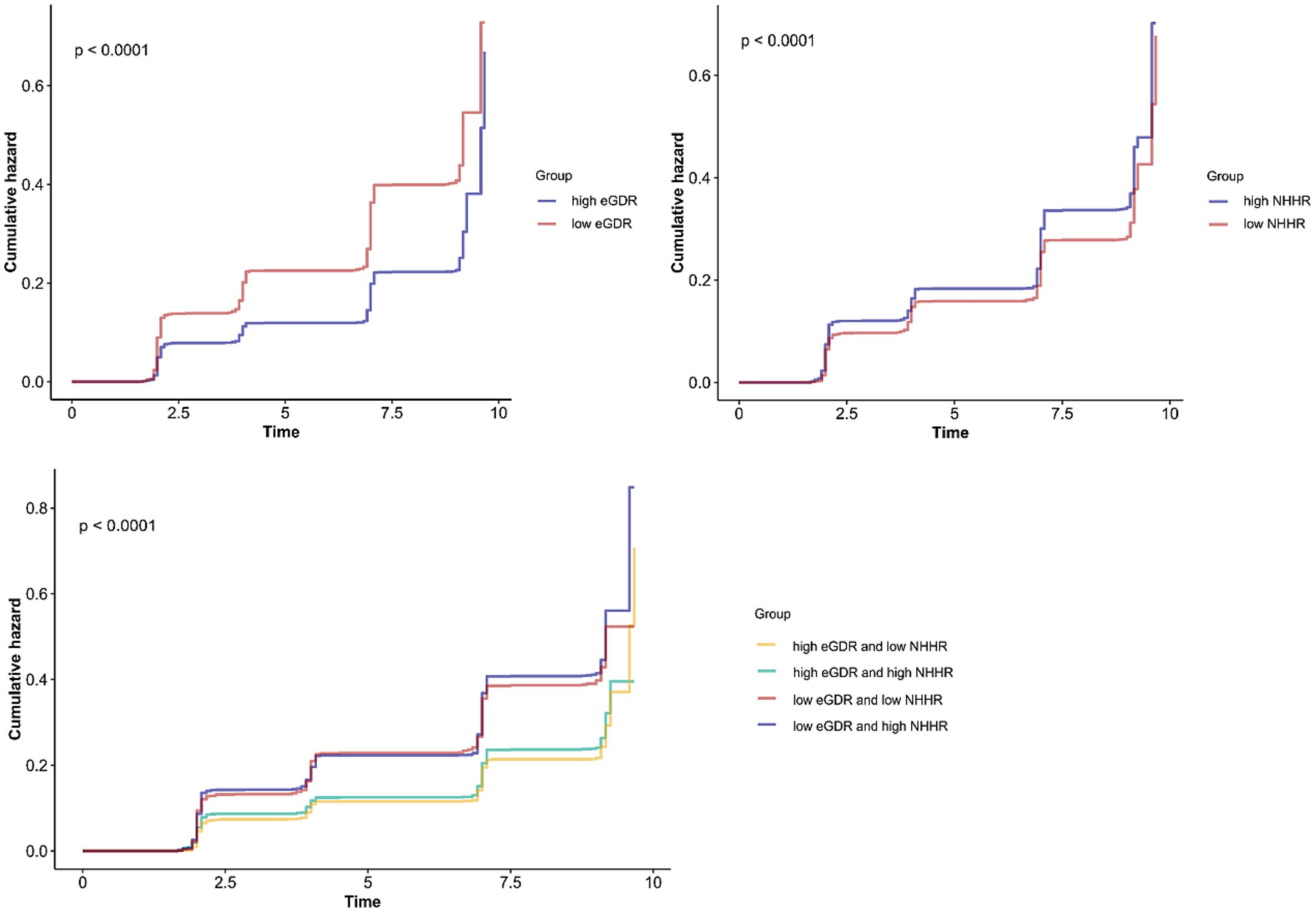
Kaplan–Meier curves for CVD incidence according to combined eGDR and NHHR groups. eGDR, estimated glucose disposal rate; NHHR, non-high-density lipoprotein cholesterol to high-density lipoprotein cholesterol ratio.

**Figure 3:**
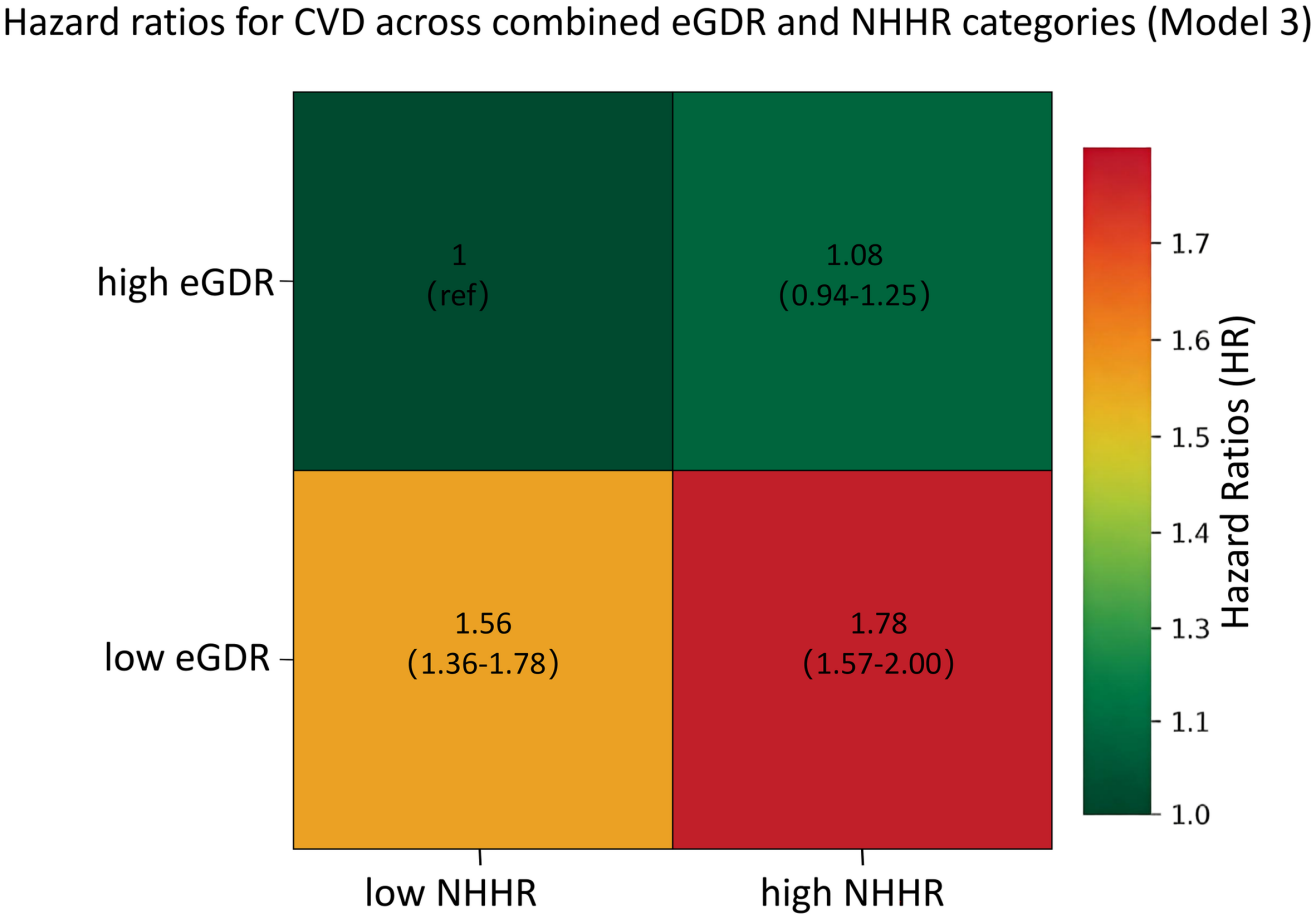
Hazard ratio heatmap for CVD risk by combined eGDR and NHHR phenotypes. Colors represent the magnitude of HR (green: lowest, red: highest). eGDR, estimated glucose disposal rate; NHHR, non-high-density lipoprotein cholesterol to high-density lipoprotein cholesterol ratio.

During a median follow-up period of 9 years, we identified 1,963 participants who developed incident CVD. The cumulative incidence of CVD varied significantly among the four groups, as shown in the Kaplan–Meier curve (log-rank test *p* < 0.0001), with the lowest incidence in the high eGDR and low NHHR group and the highest in the low eGDR and high NHHR group (Figure 2). Table 2 presents the results of the multivariable Cox proportional hazards regression analysis. In Model 3, the high eGDR and high NHHR group had no statistically significant increase in CVD risk (HR = 1.08, 95% CI 0.94–1.25, *p* = 0.283). In contrast, the risk in the low eGDR and low NHHR group was 56% higher (HR = 1.56, 95% CI 1.36–1.78, *p* < 0.001), and the risk in the low eGDR and high NHHR group was the highest, increasing by 78% (HR = 1.78, 95% CI 1.57–2.00, p < 0.001). Additionally, CVD risk increased progressively with greater metabolic burden—from the high eGDR and low NHHR group to the low eGDR and high NHHR group (P for trend < 0.001). To visually summarize this graded association, we constructed a 2 × 2 heatmap displaying the multivariable-adjusted hazard ratios (Model 3) for the four groups (Figure 3).

**Table 2:** Hazard ratios for CVD according to the combined eGDR and NHHR groups.

Group	Crude model		Model 1		Model 2		Model 3	
	HR(95%CI)	*P* value	HR(95%CI)	*P* value	HR(95%CI)	*P* value	HR(95%CI)	*P* value
High eGDR and low NHHR	1(Ref)		1(Ref)		1(Ref)		1(Ref)	
High eGDR and high NHHR	1.09 (0.94 ~ 1.26)	0.255	1.09 (0.94 ~ 1.26)	0.252	1.07 (0.93 ~ 1.24)	0.362	1.08 (0.94 ~ 1.25)	0.283
Low eGDR and low NHHR	1.77 (1.56 ~ 2.02)	<0.001	1.58 (1.39 ~ 1.8)	<0.001	1.56 (1.37 ~ 1.78)	<0.001	1.56 (1.36 ~ 1.78)	<0.001
Low eGDR and high NHHR	1.87 (1.66 ~ 2.1)	<0.001	1.76 (1.57 ~ 1.99)	<0.001	1.74 (1.54 ~ 1.95)	<0.001	1.78 (1.57 ~ 2)	<0.001
Trend.test		<0.001		<0.001		<0.001		<0.001

Model 1 adjusted for age and gender. Model 2 additionally adjusted for marital status, education, smoking, and drinking status. Model 3 further adjusted for BMI, CRP, UA, and CysC. BMI, body mass index; CI, confidence interval; CRP, C-reactive protein; CVD, cardiovascular disease; CysC, cystatin C; eGDR, estimated glucose disposal rate; HR, hazard ratio; NHHR, non-high-density lipoprotein cholesterol to high-density lipoprotein cholesterol ratio; UA, uric acid.

### 3.3 Interaction analysis

Table 3 summarizes the interaction analysis results. Incorporating the product term of eGDR and NHHR into the multivariable-adjusted Cox model did not yield a statistically significant association (*p* = 0.59). Similarly, evaluation of additive interaction showed that the 95% confidence intervals for both the relative excess risk due to interaction (RERI) and the attributable proportion (AP) encompassed 0, while that for the synergy index (SI) included 1. Taken together, these findings suggest the absence of significant multiplicative or additive interaction between eGDR and NHHR in relation to incident CVD in this population.

**Table 3:** Additive and multiplicative interaction between eGDR and NHHR on CVD risk.

Interactive indices	Interactive effects (95% CI)		
	Model 1	Model 2	Model 3
Additive effect			
RERI	0.1 (−0.15 ~ 0.34)	0.1 (−0.14 ~ 0.34)	0.13 (−0.11 ~ 0.38)
AP	0.05 (−0.08 ~ 0.19)	0.06 (−0.08 ~ 0.2)	0.08 (−0.06 ~ 0.21)
SI	1.14 (0.79 ~ 1.64)	1.16 (0.8 ~ 1.7)	1.21 (0.83 ~ 1.76)
Multiplicative effect	1.03 (0.85 ~ 1.23)	1.04 (0.86 ~ 1.25)	1.05 (0.87 ~ 1.27)

Model 1 adjusted for age and gender. Model 2 additionally adjusted for marital status, education, smoking, and drinking status. Model 3 further adjusted for BMI, CRP, UA, and CysC. AP, attributable proportion; BMI, body mass index; CI, confidence interval; CRP, C-reactive protein; CVD, cardiovascular disease; CysC, cystatin C; eGDR, estimated glucose disposal rate; HR, hazard ratio; NHHR, non-high-density lipoprotein cholesterol to high-density lipoprotein cholesterol ratio; RERI, relative excess risk due to interaction; SI, synergy index; UA: uric acid.

### 3.4 Subgroup analysis

**Figure 4:**
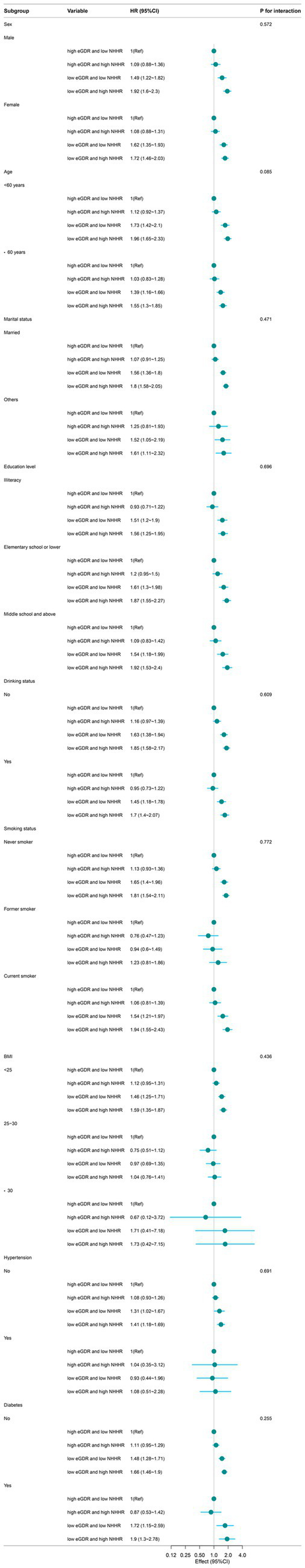
Subgroup analyses for the association between combined eGDR-NHHR phenotypes and CVD risk. BMI, body mass index; CI, confidence interval; eGDR, estimated glucose disposal rate; HR: hazard ratio; NHHR, non-high-density lipoprotein cholesterol to high-density lipoprotein cholesterol ratio.

The primary association pattern was still robust, according to the stratified analysis of various subgroups (shown in Table 4 and the forest plot in Figure 4). In particular, the high eGDR and high NHHR group consistently did not exhibit a statistically significant risk increase, whereas the low eGDR and high NHHR group maintained the highest CVD risk across all subgroups, followed by the low eGDR and low NHHR group. All interaction *p*-values were > 0.05, indicating no significant effect modification across subgroups.

**Table 4:** Subgroup analysis for the association of the eGDR and NHHR with CVD risk.

Variables	High eGDR and low NHHR	High eGDR and high NHHR	Low eGDR and low NHHR	Low eGDR and high NHHR	P for interaction
Gender, *n* (%)					0.572
Male	Ref	1.09(0.88 ~ 1.36)	1.49 (1.22 ~ 1.82)	1.92 (1.6 ~ 2.3)	
Female	Ref	1.08 (0.88 ~ 1.31)	1.62 (1.35 ~ 1.93)	1.72 (1.46 ~ 2.03)	
Age (years)					0.085
<60	Ref	1.12 (0.92 ~ 1.37)	1.73 (1.42 ~ 2.1)	1.96 (1.65 ~ 2.33)	
≥60	Ref	1.03 (0.83 ~ 1.28)	1.39 (1.16 ~ 1.66)	1.55 (1.3 ~ 1.85)	
Marital status, *n* (%)					0.471
Married	Ref	1.07 (0.91 ~ 1.25)	1.56 (1.36 ~ 1.8)	1.8 (1.58 ~ 2.05)	
Others	Ref	1.25 (0.81 ~ 1.93)	1.52 (1.05 ~ 2.19)	1.61 (1.11 ~ 2.32)	
Education level, *n* (%)					0.696
Illiteracy	Ref	0.93 (0.71 ~ 1.22)	1.51 (1.2 ~ 1.9)	1.56 (1.25 ~ 1.95)	
Elementary school or lower	Ref	1.2 (0.95 ~ 1.5)	1.61 (1.3 ~ 1.98)	1.87 (1.55 ~ 2.27)	
Middle school and above	Ref	1.09 (0.83 ~ 1.42)	1.54 (1.18 ~ 1.99)	1.92 (1.53 ~ 2.4)	
Drinking status, *n* (%)					0.609
No	Ref	1.16 (0.97 ~ 1.39)	1.63 (1.38 ~ 1.94)	1.85 (1.58 ~ 2.17)	
Yes	Ref	0.95 (0.73 ~ 1.22)	1.45 (1.18 ~ 1.78)	1.7 (1.4 ~ 2.07)	
Smoking status, *n* (%)					0.772
Never smoker	Ref	1.13(0.93 ~ 1.36)	1.65(1.4 ~ 1.96)	1.81 (1.54 ~ 2.11)	
Former smoker	Ref	0.76 (0.47 ~ 1.23)	0.94 (0.6 ~ 1.49)	1.23 (0.81 ~ 1.86)	
Current smoker	Ref	1.06 (0.81 ~ 1.39)	1.54 (1.21 ~ 1.97)	1.94 (1.55 ~ 2.43)	
BMI, kg/m^2^					0.436
<25	Ref	1.12 (0.95 ~ 1.31)	1.46 (1.25 ~ 1.71)	1.59 (1.35 ~ 1.87)	
25–30	Ref	0.75 (0.51 ~ 1.12)	0.97 (0.69 ~ 1.35)	1.04 (0.76 ~ 1.41)	
≥30	Ref	0.67 (0.12 ~ 3.72)	1.71 (0.41 ~ 7.18)	1.73 (0.42 ~ 7.15)	
Hypertension, *n* (%)					0.691
No	Ref	1.08 (0.93 ~ 1.26)	1.31 (1.02 ~ 1.67)	1.41 (1.18 ~ 1.69)	
Yes	Ref	1.04 (0.35 ~ 3.12)	0.93 (0.44 ~ 1.96)	1.08 (0.51 ~ 2.28)	
Diabetes, *n* (%)					0.255
No	Ref	1.11 (0.95 ~ 1.29)	1.48 (1.28 ~ 1.71)	1.66 (1.46 ~ 1.9)	
Yes	Ref	0.87 (0.53 ~ 1.42)	1.72 (1.15 ~ 2.59)	1.9 (1.3 ~ 2.78)	

CVD, cardiovascular disease; eGDR, estimated glucose disposal rate; NHHR, non-high-density lipoprotein cholesterol to high-density lipoprotein cholesterol ratio.

### 3.5 Sensitivity analysis

**Figure 5:**
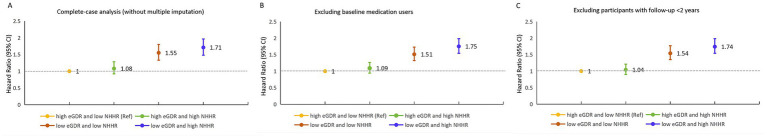
Sensitivity analyses confirming the robustness of the main findings. Hazard ratios (95% CIs) for the association between combined eGDR-NHHR groups and CVD risk under different sensitivity scenarios: **(A)** Complete-case dataset; **(B)** excluding participants using glucose-lowering, antihypertensive, or lipid-lowering drugs at baseline; **(C)** excluding participants with follow-up <2 years. The reference group is high eGDR and low NHHR (HR = 1.00). The results remained consistent with the primary analysis. CI, confidence interval; eGDR, estimated glucose disposal rate; NHHR, non-high-density lipoprotein cholesterol to high-density lipoprotein cholesterol ratio.

We conducted sensitivity analyses (detailed in Methods) to evaluate the research findings’ robustness. As shown in Figure 5, the outcomes aligned with the main findings, confirming the robustness of the association between the eGDR-NHHR joint classification and CVD risk.

### 3.6 Reclassification improvement analysis

As shown in Table 5, the combined model (eGDR+NHHR) significantly improved risk reclassification compared with NHHR alone (continuous NRI = 0.180, 95% CI 0.152–0.209, *p* < 0.001; IDI = 0.031, 95% CI 0.023–0.040, p < 0.001). In contrast, compared with eGDR alone, the combined model did not show a statistically significant improvement (continuous NRI = 0.025, 95% CI -0.003–0.050, *p* = 0.084; IDI = 0.000, 95% CI 0.000–0.001, *p* = 0.248).

**Table 5:** Improvement in reclassification for 9-year CVD risk prediction by the combined model (eGDR+NHHR) compared with single indicators.

Reference model	NRI (continuous) (95% CI)	P value	IDI (95% CI)	*p* value
eGDR	0.025 (−0.003–0.050)	0.084	0 (0–0.001)	0.248
NHHR	0.180 (0.152–0.209)	<0.001	0.031 (0.023–0.040)	<0.001

Prediction time horizon was 9 years. NRI (continuous) denotes the continuous net reclassification improvement; IDI denotes the integrated discrimination improvement. Confidence intervals and *p* values were calculated using bootstrap resampling (500 replicates).

## 4 Discussion

This study quantified the joint contribution of insulin sensitivity (estimated by eGDR) and atherosclerotic lipid profile (represented by NHHR) to CVD incidence among community-dwelling middle-aged and elderly participants from a population-based Chinese cohort. The main findings were as follows: First, insulin resistance strongly predicts CVD incidence, as evidenced by a 56% higher risk even among individuals with relatively low NHHR (the low eGDR and low NHHR group, HR = 1.56). Second, among individuals with preserved insulin sensitivity, elevated NHHR did not lead to significantly higher CVD risk (the high eGDR and high NHHR group, HR = 1.08), suggesting that in a state of excellent insulin sensitivity, isolated lipid abnormalities may not directly translate into an increase in the short-term CVD risk. Third, individuals with high NHHR and insulin resistance were most at risk for CVD (the low eGDR and high NHHR group, HR = 1.78) that approximated the additive effect of the two risk factors independently, thereby constituting a classic “dual metabolic burden.” Fourth, this insulin resistance-dominated risk gradient pattern was strong and consistent across subgroups.

Our study’s findings also indicate that, when determining the risk of CVD, insulin resistance may be more fundamental than lipid profiles. Insulin resistance was linked to a 56% increase in CVD risk even with a relatively low NHHR. Furthermore, the NRI and IDI analyses demonstrated that the addition of eGDR to NHHR significantly improved reclassification (*p* < 0.001), confirming that the combined model improves stratification. In contrast, adding NHHR to eGDR did not show any significant improvement (*p* = 0.084). This suggests that eGDR alone is a strong enough predictor, and NHHR contributes little when eGDR is already known. This asymmetric pattern highlights the dominant role of insulin resistance (reflected by eGDR) in CVD pathogenesis. This strongly implies that insulin resistance is a powerful risk factor independent of the particular lipid profile (as defined by NHHR), and may also be more predictive than conventional lipid indicators. This conclusion is in line with the general trend in the literature. For example, Yuan et al. (27) demonstrated that eGDR was an independent and robust predictor of cardiovascular mortality, with each unit decrease corresponding to a 12% increase in risk (sHR 0.88, 95% CI 0.85–0.91); even in a low-inflammatory setting, isolated insulin resistance carried an 80% higher risk of cardiovascular death (HR 1.80, 95% CI 1.34–2.41). Similarly, in another CHARLS cohort comprising 5,514 adults, the TG/HDL-C ratio showed a weaker, non-significant result (HR per SD = 1.007, 95% CI 0.943–1.082, *p* = 0.26), while the TyG index was significantly associated with the risk of incident CVD after multivariable adjustment (HR per SD = 1.176, 95% CI 1.030–1.343, *p* = 0.02) (28). It suggests that for incident CVD in this population, the indicators reflecting insulin resistance (such as the TyG index) may have better predictive value than traditional lipid ratios (e.g., TG/HDL-C). Mechanistically, low eGDR captures an insulin-resistant condition that impacts not only metabolic tissues (liver, muscle, and adipose) but also the arterial wall, resulting in vascular insulin resistance (29). Physiologically, insulin induces the PI3K/Akt/eNOS pathway in blood vessels, which results in the production of nitric oxide (NO), an important molecule for the preservation of arterial relaxation and endothelial integrity. When endothelial cells become insulin-resistant, this signaling axis is impaired, production of NO decreases, and endothelial dysfunction ensues—an early step in atherogenesis (29, 30). Insulin resistance further upregulates adhesion molecules such as VCAM-1 and ICAM-1 on the vascular endothelium, favoring adhesion and infiltration of inflammatory cells into the arterial wall (29, 31). Hyperglycemia-induced overproduction of reactive oxygen species (ROS), together with vascular insulin resistance, further exacerbates this pro-inflammatory state via activation of NF-κB and NLRP3 inflammasome pathways (32). Moreover, the pancreas secretes more insulin to compensate for reduced insulin action in peripheral tissues, which results in hyperinsulinaemia. This excess insulin in the context of insulin resistance directly stimulates vascular smooth muscle cell proliferation, lipid accumulation and pro-inflammatory cytokine release, thus accelerating atherosclerosis (33, 34). Together, these mechanisms explain why low eGDR remains a strong predictor of CVD even in the presence of a relatively favourable lipid profile (as in our low eGDR and low NHHR group, HR = 1.56, *p* < 0.001), and why the combination of insulin resistance and atherogenic dyslipidaemia confers the highest risk (HR = 1.78, *p* < 0.001).

Interestingly, an elevated NHHR was not linked to a significant increase in CVD risk in people with preserved insulin sensitivity. This suggests that, in addition to improving lipid clearance and utilization (35–37), the maintained insulin sensitivity probably mitigates the atherogenic potential of dyslipidemia via the anti-inflammatory and vascular-protective effects of insulin, which counteract the vascular damage induced by dyslipidemia (38, 39). This phenomenon is similar in mechanism to the concept of “metabolically healthy obesity,” which suggests that the preserved insulin sensitivity may lessen the cardiometabolic risks associated with obesity (40, 41). But the development of insulin resistance disrupts this condition. It triggers the fundamental pathological pathways listed above, such as oxidative stress, endothelial dysfunction, and chronic inflammation (42–44), coupled with directly aggravating lipid disorders [e.g., by encouraging the secretion of VLDL and hindering lipoprotein clearance (35)]. At this point, the pathological effects of dyslipidemia and insulin resistance combine, creating a “double burden” that accelerates the progression of atherosclerosis. This is supported by a previous study. In the Framingham Offspring Study cohort, insulin resistance was shown to significantly amplify the coronary heart disease risk conferred by the atherosclerotic lipid profile (45). This finding has important implications for the clinical interpretation of lipid results: for insulin-sensitive patients, isolated elevation of NHHR may require comprehensive assessment in conjunction with other risk factors, while for insulin-resistant patients, even if their NHHR is at a relatively low level, they should be considered as high-risk individuals and need active intervention to improve insulin sensitivity. Therefore, our results reinforce a core view: beyond its role as an independent risk factor, insulin resistance functions as a key “amplifier” that determines whether other metabolic risk factors (such as dyslipidemia) can ultimately lead to clinical events.

Nevertheless, we cannot exclude alternative explanations for the null finding in the high eGDR and high NHHR group. First, although this subgroup included 1,215 participants and the 95% confidence interval (0.94–1.25) excludes a moderate-to-large effect (e.g., HR > 1.5), the study may still lack sufficient power to detect a very small increase in risk (e.g., HR = 1.05–1.10). Second, the 9-year follow-up period might be insufficient for isolated dyslipidemia (high NHHR without insulin resistance) to manifest as clinical CVD events. In support of this possibility, the Framingham Offspring Study, which had a longer follow-up of 11.6 years, detected a significant 1.4- to 1.7-fold increased CVD risk in participants with metabolic syndrome but without insulin resistance—a phenotype similar to our high eGDR and high NHHR group (45). Thus, a longer observation period might reveal an elevated risk in this group. Future studies with extended follow-up are warranted to clarify this issue.

The lack of a significant statistical interaction between eGDR and NHHR suggests that their effects on cardiovascular disease risk are largely independent and additive. This characteristic does not undermine the clinical value of their combined assessment; on the contrary, it helps us establish a clear risk stratification model (as shown in our four groups). Such a model excels not only at precisely identifying, for intensive management, the highest-risk individuals experiencing a “dual metabolic burden”—insulin resistance and dyslipidemia—but also at identifying potentially high-risk individuals with insulin resistance but relatively low NHHR (i.e., “isolated insulin resistance”), a group that might be overlooked in lipid-centric assessment paradigms. This emphasizes the importance of assessing insulin sensitivity along with the lipid profile in the evaluation.

The study has the following methodological design advantages: a nationally representative sample ensures the reliability of the results; the combination of a prospective design and long-term follow-up effectively reduces bias, minimizes missed diagnoses, and ensures the integrity of the outcome events; and extensive control of confounding factors and detailed effect modification analysis significantly improve the robustness of effect estimation. There are certain limitations of this study, though. First, the CVD outcomes are based on self-reporting. Validation studies in CHARLS have shown that self-reports of chronic conditions have high specificity but low sensitivity (46, 47). A validation study specifically targeting incident CVD estimated that self-reported CVD has a sensitivity of approximately 51% and a specificity exceeding 98% (48). Since outcome underreporting is likely non-differential across exposure groups, the resulting bias would compress the hazard ratio estimates toward the null (49). Therefore, the results of this study should be interpreted as conservative estimates of the risk association. Second, although CHARLS required an overnight fast (>92% compliance), we could not verify individual adherence and thus cannot completely exclude residual confounding from non-fasting status. However, accumulating evidence indicates that non-fasting lipid measurements provide comparable cardiovascular risk prediction to fasting samples (50, 51), and the NHHR ratio is relatively stable, which partially mitigates this concern. Third, the observational design prevents causal inference, and residual confounding (e.g., diet, physical activity, or family history) may remain. Future studies with Mendelian randomization or other causal inference approaches are required to confirm the associations observed here. Fourth, CVD outcomes were defined as a composite endpoint based on self-reported heart disease or stroke, and specific subtypes (e.g., coronary heart disease, myocardial infarction, ischemic versus hemorrhagic stroke) were not distinguished. Further studies are needed to confirm the predictive performance of the combined marker in each subtype. Fifth, the study sample was restricted to Chinese adults aged 45 years or older, and the conclusions should be interpreted with caution when extrapolated to other age groups, ethnic groups, and regions. Sixth, in this study we only tested eGDR which has been shown to perform better in Chinese populations in previous studies. This is a defensible choice methodologically, but the absence of direct comparisons of eGDR and these other metrics in the same cohort is a limitation. Future studies must therefore include head-to-head evaluations.

In addition, the increasing importance of polygenic risk scores (PRS) as a useful tool to improve cardiovascular risk stratification, especially in people with dyslipidemia, is also worth mentioning. Bosco and colleagues (52) show that PRS could aid in diagnosing non-monogenic hypercholesterolemia, better stratifying CVD risk beyond LDL cholesterol, and shaping individualized lipid-lowering strategies. For instance, a large-scale study developed and validated an integrated PRS for eight cardiovascular diseases and observed that, for severe hypercholesterolemia, a high PRS was associated with more than a fourfold higher risk (OR 4.1, 95% CI 3.7–4.5), underlining its ability to identify individuals at high risk beyond traditional lipid parameters (53). Although a recent clinical consensus statement from the European Society of Cardiology further supports that PRS can capture lifelong genetically mediated risk and may refine CVD prediction (54), more evidence is still needed for routine clinical application of PRS. Whereas our study was based on readily available clinical parameters, incorporation of such genetic information is an exciting future area of research that could further improve the precision of risk assessment in this population.

## 5 Conclusion

This CHARLS-based cohort study provides evidence that insulin resistance characterized by low eGDR is a powerful predictor of CVD risk, independent of the level of NHHR. The combined assessment of eGDR and NHHR can effectively distinguish individuals with gradually increasing risk across different metabolic phenotypes, especially identifying those with the “dual high-risk” characteristics of both insulin resistance and atherogenic dyslipidemia, as well as potential high-risk individuals with isolated insulin resistance. Given that both of these indicators can be easily obtained through routine physical examinations and blood tests, this combined assessment model provides a practical tool for simple and efficient early screening and stratification of CVD risk in primary medical care and health management. It can guide targeted lifestyle interventions and drug treatments, thereby optimizing the primary prevention strategy for CVD.

## Data Availability

The datasets analysed during the current study are available from the China Health and Retirement Longitudinal Study (CHARLS) repository. Researchers can access the data by registering and submitting a request through the official CHARLS website at http://charls.pku.edu.cn/en.
